# The Effects on the Femoral Cortex of a 24 Month Treatment Compared to an 18 Month Treatment with Teriparatide: A Multi-Trial Retrospective Analysis

**DOI:** 10.1371/journal.pone.0147722

**Published:** 2016-02-09

**Authors:** Tristan Whitmarsh, Graham M. Treece, Andrew H. Gee, Kenneth E. S. Poole

**Affiliations:** 1 University of Cambridge, Department of Engineering, Trumpington Street, Cambridge, CB2 1PZ, United Kingdom; 2 University of Cambridge, Department of Medicine, Addenbrooke’s Hospital, Cambridge, CB2 0QQ, United Kingdom; David Geffen School of Medicine, UNITED STATES

## Abstract

**Background:**

Teriparatide (TPTD) is an anabolic agent indicated for the treatment of severely osteoporotic patients who are at high risk of fragility fractures. The originally approved duration of TPTD treatment in several regions, including Europe, was 18 months. However, studies of areal bone mineral density (aBMD) showed additional benefit when treatment is continued beyond 18 months, and the drug is currently licenced for 24 months. Improvements in cortical structure at the proximal femur have already been shown in patients given TPTD for 24 months using quantitative computed tomography (QCT). Here, we investigate whether cortical and endocortical trabecular changes differ between an 18- and 24-month treatment.

**Methods:**

Since an 18- versus 24-month TPTD study using QCT has not been conducted, we studied combined QCT data from four previous clinical trials. Combined femoral QCT data from three 18-month TPTD studies (‘18-month group’) were compared with data from a fourth 24-month trial (‘24-month group’). Cortical parameters were measured over the entire proximal femur which allowed for a comparison of the mean changes as well as a visual comparison of the colour maps of changes after 18 and 24 months TPTD.

**Results:**

For both the combined 18-month group and the 24-month group, overall cortical thickness and endocortical trabecular density increased, while overall cortical bone mineral density decreased. While the changes in the 24-month group were of greater magnitude compared to the 18-month group, the differences were only significant for the endocortical trabecular density (ECTD), corrected for age, weight, femoral neck T-score, total hip T-score and the baseline mean ECTD.

**Conclusion:**

Although the combination of data from different clinical trials is not optimal, these data support the concept that the duration of TPTD in the 18–24 month phase is of clinical relevance when considering improvement in hip structure.

## Introduction

Osteoporosis is an age-related disease characterized by a deterioration of the bone micro-structure through porosity and thinning, which results from an imbalance between bone formation and resorption. This causes the bone to become more fragile and increases the risk of fracture.

In 2002 the FDA approved Teriparatide (TPTD) for the treatment of postmenopausal women with osteoporosis at high risk of fracture for a duration of 24 months. In a large prospective study TPTD was shown to increase hip and spine areal bone mineral density (aBMD) and reduce vertebral and nonvertebral fracture incidence within a mean treatment duration of 18 months [1]. Other studies show that improvements in aBMD at the proximal femur by TPTD occur predominantly beyond the 18-month period [2]. A two-year prospective study on the effects of TPTD treatment with or without a previous antiresorptive therapy [3] indicates a steady increase in total hip aBMD and femoral neck aBMD after the first 6 months. Between the 18 and 24 month time point, aBMD increases were large and statistically significant for both pre-treated and treatment naive subjects. Moreover, in the anti-resorptive pretreated subjects, the increases in aBMD at the total hip and femoral neck were approximately twice as large after a 24-month TPTD treatment compared to an 18-month treatment. These studies, however, examine the aBMD only. These projected density measurements cannot distinguish trabecular from cortical bone and provide limited information on the structural changes.

In an ancillary study of the same clinical trial, quantitative computed tomography (QCT) scans were acquired at 6, 12 and 24 months [4]. The 18 month time point was not included due to limitations in the radiation dose admissible to the patient. No clinical trial has ever been performed which includes QCT scan acquisitions on the effects of an additional 6-month TPTD treatment after a previous 18-month treatment, and QCT scan data for such analysis is hence not available. However, as the studies on aBMD indicated, the treatment of an additional 6 months might provide additional benefits to the patient which outweigh any adverse effects or added cost.

To investigate cortical and trabecular responses, in this retrospective analysis, we combined the QCT data from three clinical trials of an 18-month TPTD treatment. We then compared this dataset with QCT data from a fourth clinical trial which examined changes in the proximal femur after 24 months of TPTD therapy. In addition, we performed a statistical parametric mapping analysis on the data, which allowed us to visualise where statistically significant changes occurred on the proximal femur for the 18- and 24-month treatments.

## Materials and Methods

### Data

This study combines the data from four clinical trials where patients received 20 μg TPTD by daily subcutaneous injection and hip QCT scans were available. For examining the effects of an 18-month treatment by TPTD, three datasets were used, denoted GHDJ, GHBM and GHCM:

- The GHDJ dataset, resulting from the Micro-MRI US trial (ClinicalTrials.gov number NCT00557310), includes 24 osteoporotic women with a mean age of 60.9 ± 7.4 years who received 18 months of TPTD therapy [5].
- The GHBM dataset results from the FACT study (ClinicalTrials.gov number NCT02416271) which compared a TPTD treatment with an alendronate (ALN) treatment [6]. From this dataset we incorporate only the TPTD cohort. This includes 19 postmenopausal women with a mean age of 66.9 ± 7.6 years.
- The GHCM (Add vs. Switch) dataset results from a study (ClinicalTrials.gov number NCT00079924) examining the effects of switching to or adding an 18-month TPTD treatment to a previous treatment by ALN or raloxifene (RLX). From this dataset we include only the subjects whereby a previous ALN or RLX treatment was switched to a TPTD treatment. In this dataset, the QCT scans that were acquired after switching to TPTD were used as baseline with follow-up QCT scans after the 18-month TPTD treatment. This dataset contains 65 postmenopausal women with a mean age of 68.9 ± 8.4 years. The details of this clinical trial are reported in [7] and [8].

These three datasets were combined into a single ‘18-month group’ which consists of 108 subjects with a mean age of 66.8 ± 8.6 years who all received TPTD for 18 months.

The full set of baseline characteristics are presented in Table 1. Significances of the differences of all baseline parameters are assessed by ANOVA. Due to the fact that these three datasets result from different clinical trials, there are some statistically significant differences (p<0.05) in the baseline parameters, specifically the age, weight and T-score at the total hip. In addition the baseline mean cortical thickness (CTh) and cortical bone mineral density (CBMD) are statistically significantly different between the groups.

**Table 1:** Baseline characteristics (mean ± SD) with the significances of the differences assessed by ANOVA.

	18-months					24-months	
	GHDJ (n = 24)	GHBM (n = 19)	GHCM (n = 65)	ANOVA (p-value)	Combined (n = 108)	EUROFORS (n = 64)	ANOVA Combined/EUROFORS (p-value)
Age (years)	60.9 ± 7.4	66.9 ± 7.6	68.9 ± 8.4	<0.001	66.8 ± 8.6	67.6 ± 6.9^1^	0.537^1^
Weight (kg)	56.4 ± 9.1	63.0 ± 11.1	63.1 ± 9.0	0.010	61.6 ± 9.7	64.1 ± 10.8	0.125
Height (cm)	158.7 ± 7.6	155.8 ± 6.6	159.5 ± 5.9	0.095	158.7 ± 6.5	157.7 ± 7.2^2^	0.365^2^
T-score femoral neck	-2.5 ± 0.7	-2.2 ± 0.7	-2.4 ± 0.6	0.269	-2.3 ± 0.6	-2.4 ± 0.9	0.381
T-score total hip	-1.8 ± 0.8	-1.6 ± 0.6	-2.1 ± 0.7	0.018	-2.0 ± 0.7	-2.8 ± 0.8	<0.001
Mean CTh (mm)	1.4 ± 0.1	1.3 ± 0.1	1.3 ± 0.1	0.017	1.4 ± 0.1	1.4 ± 0.1	0.975
Mean CBMD (mg/cm^3^)	898.3 ± 28.4	941.7 ± 28.0	919.8 ± 42.2	0.001	918.9 ± 39.5	932.3 ± 50.3	0.054
Mean ECTD (mg/cm^3^)	111.2 ± 22.4	116.1 ± 26.0	104.5 ± 22.2	0.120	108.1 ± 23.2	90.3 ± 23.6	<0.001
Mean CMSD (mg/cm^2^)	130.6 ± 12.1	125.5 ± 12.1	124.1 ± 14.9	0.146	125.8 ± 14.0	127.4 ± 14.8	0.473

^1^ derived from n = 62 subjects in the EUROFORS group since for two subjects age data was not available.

^2^ derived from n = 62 subjects in the EUROFORS group since for two subjects height data was not available.

For the ‘24-month group’ we used the QCT scans resulting from the EUROFORS trial (ClinicalTrials.gov number NCT00191425). This clinical trial examined the effects of various sequential treatments after TPTD in postmenopausal woman with established osteoporosis. Results of this trial on aBMD measurements were previously reported in [9] and [3], and a subgroup study on the hip QCT data in [4]. Only those subjects were included who were switched to TPTD and continued the TPTD treatment for a duration of 24 months, yielding 64 patients with a mean age of 67.6 ± 6.9 years. The baseline characteristics of this cohort are also presented in Table 1. T-scores were computed from the QCT scans using the Mindways CTXA-Hip software (Mindways Software, Austin, TX, USA). The demographics and baseline measurements of the EUROFORS hip QCT subgroup were compared with the pooled 18-month cohort by ANOVA and indicated a significant difference in total hip T-score and endocortical trabecular density. The difference in baseline mean cortical bone mineral density was nearing significance with p = 0.054.

All scans were acquired with the Mindways calibration phantom in the field of view, allowing for the Hounsfield units to be converted to bone mineral density values. Institutional Review Board approval was obtained from each of the clinical study sites, and written informed consent was obtained from each participant.

### Cortical measurements

The cortical parameter measurement and mapping technique has been previously described [10, 11, 12] and is implemented in the software tool Stradwin (http://mi.eng.cam.ac.uk/~rwp/stradwin/). Using this software, the contours of the bone surface are semi-automatically drawn in the QCT slices. These are then processed into a triangular surface mesh on which the cortical measurements are performed. Samples are taken in the QCT volume perpendicular to the bone surface, resulting in a profile of the QCT values with an inherent smoothness. By fitting a blurred model of the cortex to the data samples, Stradwin is able to accurately estimate the CTh (mm) and the CBMD (mg/cm^3^) as well as the endocortical trabecular density (ECTD, mg/cm^3^), which is the trabecular density directly adjacent to the cortex. From the cortical thickness and CBMD, we also compute the cortical mass surface density (CMSD) as CMSD = 0.1 × CTh × CBMD, which represents the mass per unit surface area (mg/cm^2^). In this study, cortical bone mapping version 2 (CBMv2) is used, as referenced in [12].

Stradwin measures the cortical parameters at each vertex of the bone surface mesh which results in a colour coded map of the cortical parameter values. Cortical parameter maps are subsequently produced for each QCT scan which are all mapped to a canonical surface using wxRegSurf (http://mi.eng.cam.ac.uk/~ahg/wxRegSurf/) to give us a one-to-one correspondence between the cortical parameters of each femur across all four studies. This first requires a canonical shape to be registered to the individual shape by a deformable registration. After this registration, the parameters are mapped to the canonical surface by, for each vertex in the canonical mesh, finding the closest point in the individual mesh and taking the associated cortical parameter.

### Statistical analysis

Since we are performing a longitudinal study, we have the cortical maps at baseline and at the 18 or 24 month endpoint. By subtracting baseline from follow-up, we generate maps of the changes in the cortical parameters resulting from either the 18-month or 24-month treatment. Mean cortical parameter changes for each subject, and subsequently the within-group changes, were calculated with the associated significances assessed by T-tests. When comparing the differences of the mean cortical parameter changes between the three 18-month cohorts, we assess the significances by ANOVA corrected for age, weight, femoral neck T-score and total hip T-score, as well as the baseline mean cortical parameter values of the corresponding parameter. Here, a linear regression model is fitted to the data with the cortical parameter changes as the response variable and the group, age, weight, height, femoral neck T-score, total hip T-score and baseline mean parameter values as the predictor variables. ANOVA then computes the variability within the regression model and computes the F-statistics for testing the significance of the group term.

In this study, the combined 18-month dataset was compared with the 24-month GHCA dataset. At baseline significant differences were observed for the total hip T-score, as well as for the baseline mean ECTD. The significance of the differences in the cortical changes between the 18- and 24-month datasets were assessed by ANOVA tests, corrected for age, weight, height, femoral neck T-score, total hip T-score and mean baseline value of the corresponding cortical parameter.

Statistical parametric mapping produced a map showing the regional significances of the changes. The significance maps were combined with the cortical change maps whereby the regions of insignificant change (p>0.05) are indicated in grey. This cortical parameter mapping technique is explained in detail in [13].

## Results

All cortical parameter changes are presented in Table 2. For the combined 18-month TPTD treatment groups, CTh and ECTD increased, while CBMD and CMSD decreased, although not all changes were statistically significant. Corrected for the confounding factors, the differences in cortical parameter changes are not significant (based on a significance threshold of p = 0.05), although the CTh change is nearing significance with p = 0.052.

**Table 2:** Cortical parameter changes as mean (95% CI) change and the significances of the changes assessed by T-test.

	18-months					24-months	
	GHDJ (n = 24)	GHBM (n = 19)	GHCM (n = 65)	ANOVA^1^ (p-value)	Combined (n = 108)	EUROFORS (n = 64)	ANOVA^1^^,^^2^ Combined/EUROFORS (p-value)
CTh (mm)	0.019 (-0.008 to 0.047)	0.064 (0.037 to 0.091)^‡^	0.032 (0.021 to 0.044)^‡^	0.052	0.035 (0.024 to 0.045)^‡^	0.044 (0.029 to 0.058)^‡^	0.559
CBMD (mg/cm^3^)	-19.5 (-30.5 to -8.5) ^‡^	-41.9 (-62.9 to -20.9) ^‡^	-23.9 (-33.6 to -14.2)^‡^	0.305	-26.1 (-33.5 to -18.7)^‡^	-33.1 (-41.0 to -25.1)^‡^	0.673
ECTD (mg/cm^3^)	1.7 (-3.8 to 7.1)	4.3 (0.4 to 8.2)^†^	4.2 (2.3 to 6.1)^‡^	0.520	3.6 (1.9 to 5.4)^‡^	8.8 (6.4 to 11.3)^‡^	0.004
CMSD (mg/cm^2^)	-1.3 (-4.4 to 1.8)	-0.4 (-2.6 to 1.9)	-0.8 (-2.0 to 0.5)	0.783	-0.8 (-1.9 to 0.3)	-0.8 (-2.2 to 0.6)	0.461

^1^ correction for age, weight, height, femoral neck T-score, total hip T-score and baseline of corresponding cortical parameter.

^2^ derived from n = 61 subjects in the EUROFORS group since for three subjects age or height data was not available.

^†^ significant change from baseline where p<0.05;

^‡^ significant change from baseline where p<0.01.

Both the 18-month combination group and the 24-month treatment group showed a statistically significant increase in overall CTh and ECTD with a significant decrease in CBMD. These changes are greater for the 24-month cohort compared to the 18-month groups, although the differences are not significant for the CTh and CBMD. ECTD increases, however, are significantly greater for the 24-month compare to the 18-month group corrected for age, weight, height, femoral neck T-score, total hip T-score and baseline ECTD (p = 0.004). There were no significant changes for CMSD measurements, nor are there for any of the individual 18-month treatment groups.

**Fig 1:**
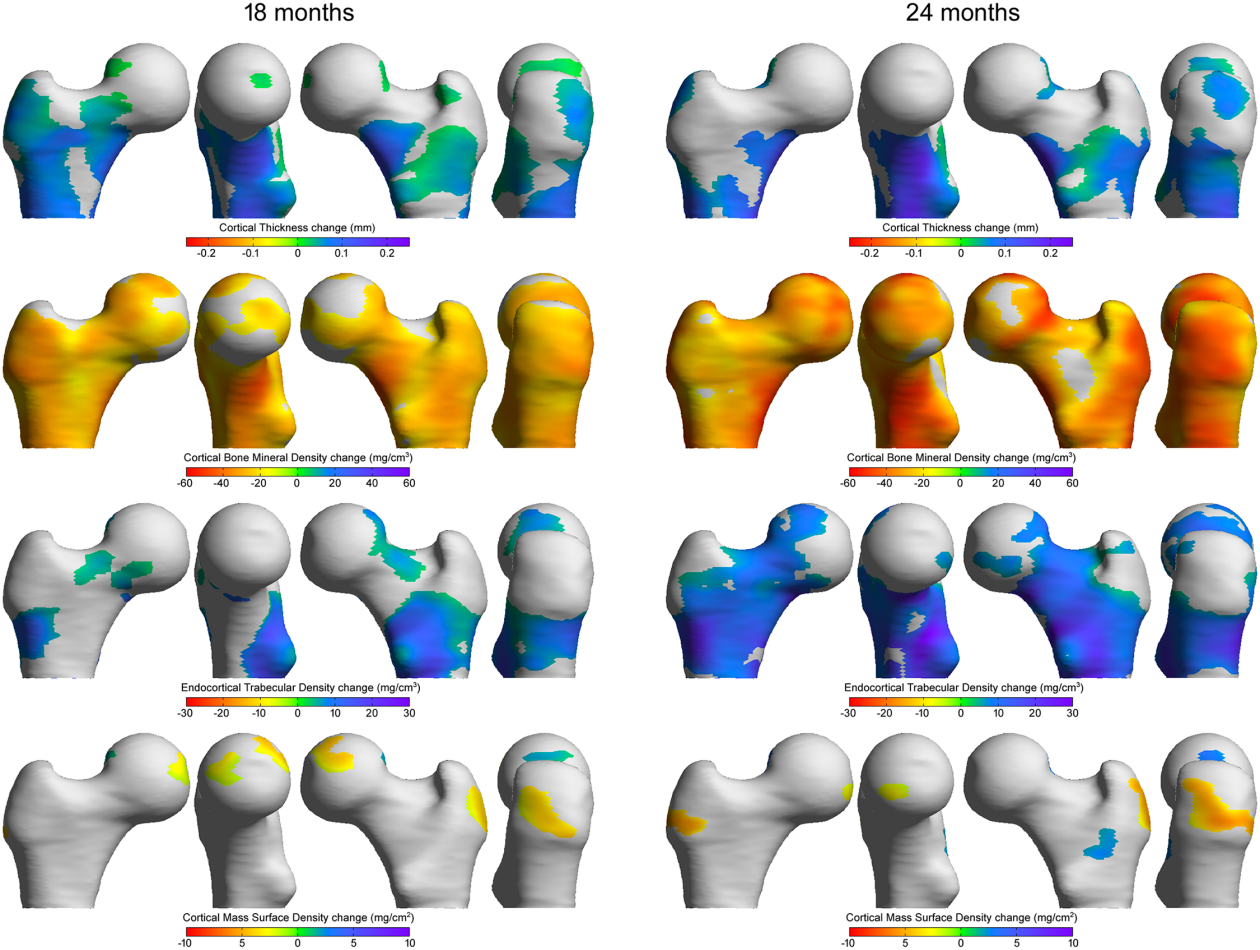
Cortical change maps (versus baseline) of the combined 18 month TPTD treatment cohort (left) and 24 month TPTD treatment cohort (right). The colours express the mean changes in the cortical parameter values. The grey regions indicate the changes to be not statistically significant (p>0.05) based on F-tests.

When examining the colour coded maps (Fig 1), large regions of significantly increased cortical thickness were evident for both the 18- and 24-month groups. Changes were predominantly observed at those regions predicted to receive high loads during walking (including the inferomedial cortex and the calcar femorale regions). For the 24-month treatment group, changes appeared to be of greater magnitude than the 18-month treatment group at corresponding locations. CBMD maps indicated large and statistically significant reductions in CBMD across most of the proximal femur, which appear to be of greater magnitude for the 24-month cohort than the 18-month cohort. Also the increase in ECTD was more pronounced for the 24-month group compared to the 18-month group with larger regions of statistical significant change. For the CMSD maps of both the 18- and the 24-month group we can see significant reductions in mass at the attachment point of the gluteus medius, the attachment point of a key muscle of locomotion, despite an increase in thickness.

## Discussion

This study combines the data from various different clinical trials to form an 18-month TPTD treatment group. Combining data from patients with different demographics is precarious and should be handled with great care. The population characteristics have been assessed for differences between the three 18-month treatment groups. This indicated that there were indeed significant differences between these populations.

The significances of the differences in cortical changes between the three groups were also assessed. Here we used a linear model to correct for age, weight, height, femoral neck T-score, total hip T-score and the corresponding baseline mean parameter value. This showed no significant difference between the 18-month groups. This indicates that, although the cortical parameter changes vary considerably between groups (Table 2), these differences can largely be accredited to the differences in the populations.

The cortical changes in the combined 18-month cohort were compared to the cortical changes from a fourth independent clinical trial with a 24 month TPTD treatment period. The cortical changes were compared for the significance in difference with the combined 18-month cohort by ANOVA tests corrected for age, weight, height, femoral neck T-score, total hip T-score and the corresponding baseline mean parameter value. Only the changes in ECTD remain significantly greater for the 24-month group compared to the 18-month group. This might indicate persistent bone forming activity even after the 18-month time point. This is clinically relevant since a recent study showed ECTD to be a strong predictor of hip fractures [14]. Improving ECTD is therefore a valid target when considering strategies to reduce hip fractures. It should however be noted that, although the changes in ECTD remain significantly different when corrected for baseline measurements, ECTD was not balanced at baseline and caution is therefore needed when interpreting these results.

As in our previous studies [11, 15, 16], TPTD led to widespread thickening of the cortex with increasing endocortical trabecular density, while there was very little change in CMSD as well as a widespread reduction in CBMD. Similarly, Borgreffe et al. [4] found with the EUROFORS dataset, examined with the Mindways QCT-Pro technique, that cortical cross sectional area increased and cortical vBMD decreased after 24 months. An explanation for these findings has been provided in [16] where new bone deposits were shown to be measured as either an increase in cortical thickness with a greater porosity (i.e. a decreased CBMD) or an increased ECTD. While in previous studies cortical changes were described as percentages with respect to baseline, the differences in the mean cortical parameters at baseline for the different trials makes it more appropriate to report the magnitude of the changes in this study.

Previous studies indicate that a prior antiresorptive treatment might blunt areal BMD increases by TPTD, depending on the type of antiresorptive [17]. Both the 18-month group and the 24-month group include pre-treatment naive as well as antiresorptive pre-treated patients, but of varying types and treatment durations. Thus, although some of the changes seen in this study might be affected by pre-treatment regimes, the statistical analysis cannot be adequately corrected for these pre-treatments due to the great variability.

The main limitation of this study is the fact that the trial group and the time point are confounded. We do not know whether the 24-month group had the same cortical changes at the 18 month time point as the pooled 18-month study group, and there is no common therapy time point that can be adjusted against. Furthermore, there are substantial limitations in combining study subjects from different clinical trials, performed at different locations and using different populations, even when the study drug is the same. The combination of different trials prevents us from making firm conclusive statements about the outcome. However, we do see a greater endocortical trabecular response in the 24-month group compared to the combined 18-month group, where the increases of the 24-month group were statistically significantly greater than the 18-month groups even when corrected for the demographics and the mean ECTD at baseline. The magnitude of the changes for the other parameters appear greater with 24 months treatment, but do not achieve statistical significance after correcting for demographics and baseline mean differences. As the ECTD has recently been shown to be a strong predictor for hip fractures, an additional 6-month TPTD treatment might reduce the risk of hip fracture by increasing the trabecular bone, which potentially outweighs possible adverse effects and added costs. The increased ECTD is also reflected by the cortical maps which show greater changes for the 24-month treatment compare to the combined 18-month treatment group. In addition, the geometrical spread of changes is very similar between the two groups, which is some indication of the validity of comparing this data. Although no firm conclusions can be made from this multi-trial retrospective analysis, this study does suggest that a 6-month treatment with TPTD after a previous 18-month treatment could enhance trabecular bone structure of the hip.
